# Variants in *NEB* and *RIF1* genes on chr2q23 are associated with skeletal muscle index in Koreans: genome-wide association study

**DOI:** 10.1038/s41598-021-82003-y

**Published:** 2021-03-05

**Authors:** Kyung Jae Yoon, Youbin Yi, Jong Geol Do, Hyung-Lae Kim, Yong-Taek Lee, Han-Na Kim

**Affiliations:** 1 Department of Physical & Rehabilitation Medicine, Kangbuk Samsung Hospital, Sungkyunkwan University School of Medicine, 29 Saemunan-ro, Jongno-gu, Seoul, 03181 Republic of Korea; 2 Medical Research Institute, Kangbuk Samsung Hospital, Sungkyunkwan University School of Medicine, 29 Saemunan-ro, Jongno-gu, Seoul, 03181 Republic of Korea; 3 Biomedical Institute for Convergence at SKKU, Sungkyunkwan University School of Medicine, Suwon, Republic of Korea; 4 Department of Clinical Research Design & Evaluation, SAIHST, Sungkyunkwan University, Seoul, Republic of Korea; 5 Department of Biochemistry, College of Medicine, Ewha Womans University, Seoul, Republic of Korea

**Keywords:** Genetics, Health care, Medical research

## Abstract

Although skeletal muscle plays a crucial role in metabolism and influences aging and chronic diseases, little is known about the genetic variations with skeletal muscle, especially in the Asian population. We performed a genome-wide association study in 2,046 participants drawn from a population-based study. Appendicular skeletal muscle mass was estimated based on appendicular lean soft tissue measured with a multi-frequency bioelectrical impedance analyzer and divided by height squared to derive the skeletal muscle index (SMI). After conducting quality control and imputing the genotypes, we analyzed 6,391,983 autosomal SNPs. A genome-wide significant association was found for the intronic variant rs138684936 in the *NEB* and *RIF1* genes (β = 0.217, *p* = 6.83 × 10^–9^). These two genes are next to each other and are partially overlapped on chr2q23. We conducted extensive functional annotations to gain insight into the directional biological implication of significant genetic variants. A gene-based analysis identified the significant *TNFSF9* gene and confirmed the suggestive association of the *NEB* gene. Pathway analyses showed the significant association of *regulation of multicellular organism growth* gene-set and the suggestive associations of pathways related to skeletal system development or skeleton morphogenesis with SMI. In conclusion, we identified a new genetic locus on chromosome 2 for SMI with genome-wide significance. These results enhance the biological understanding of skeletal muscle mass and provide specific leads for functional experiments.

## Introduction

Since Rosenberg first coined the term sarcopenia in 1989^1^, clinicians have been increasingly interested in skeletal muscle mass and strength, considering that these factors are associated with functional performance, metabolism, and even survival^2–4^. Low skeletal muscle mass is related to metabolic problems including insulin resistance and cardiovascular risk^5,6^ not only in the elderly but also in the general population (including young adults).

A recent European and Asian consensus provided the definition of sarcopenia^7,8^, in which skeletal muscle mass was estimated with dual-energy X-ray absorptiometry (DEXA) or bioelectrical impedance analysis (BIA). BIA is one of the most useful methods for estimating the volume of skeletal muscle mass, especially given the inexpensiveness and reproducibility of this technique^9^. As skeletal muscle mass is associated with body size, the European and Asian working groups for sarcopenia adjusted the skeletal muscle mass with the height squared^7,8^.

Skeletal muscle mass is known to have a strong genetic determination, with a heritability of over 50%^10^. However, few studies have reported a genetic predisposition for skeletal muscle mass. In some studies^11–14^, a genome-wide association study (GWAS) was performed for lean body mass, of which the main component is skeletal muscle. Although recent technical advances have allowed a GWAS to be used as an unbiased method for screening the whole human genome for novel genes for skeletal muscle mass, most have been conducted exclusively in Caucasian populations^14–17^ and the results were not consistent. In contrast, a relatively small number of studies have been reported in Asian populations^11,18^. Ethnic differences in skeletal muscle mass are known to exist^19^. Previous studies reported that Asian people generally had less muscle mass than Caucasians^20,21^.

While previous studies mainly focused on identifying candidate genes, the gene- and gene set-based approaches allows GWAS results to be integrated with genes in predefined human databases, offering a complementary approach to data interpretation. A gene-based GWAS on skeletal muscle mass was performed previously, but it was also a study in Caucasians^13^.

We conducted a GWAS using single variants for skeletal muscle mass, which was represented by the skeletal muscle index (SMI, skeletal muscle mass divided by height squared) and also gene- and gene set-based analyses using the results of the GWAS in a Korean population. The purpose of this study was to identify the associations of specific genetic variations with the SMI and elucidate the biological mechanisms through functional annotation.

## Results

### Subject demographics

The study sample was comprised of 1,150 men and 896 women with a mean age of 39.3 years (standard deviation [SD] 8.9), ranging from 20 to 69 years (Table 1). The mean skeletal muscle index (SMI) value was 9.7 kg/m^2^ (SD 1.5) in men and 9.3 kg/m^2^ (SD 1.5) in women. The average body mass index (BMI) was 24.4 kg/m^2^ (SD 2.8) in men and 21.6 kg/m^2^ (SD 2.8) in women (Table 1).

**Table 1:** Baseline characteristics of the study population.

Characteristics	Men (n = 1150)	Women (n = 896)	Total (n = 2046)
Age (years)	39.9 (8.9)	38.7 (8.7)	39.3 (8.9)
Height (cm)	173.2 (5.7)	160.7 (5.1)	167.7 (8.2)
Weight (kg)	73.4 (9.6)	55.8 (7.7)	65.7 (12.4)
Body mass index (kg/m^2^)	24.4 (2.8)	21.6 (2.8)	23.2 (3.2)
Skeletal muscle index (kg/m^2^)^a^	9.7 (1.5)	9.3 (1.5)	9.5 (1.5)
Skeletal muscle mass (kg)	31.7 (3.5)	21.0 (2.5)	27.0 (6.1)
Muscle mass (kg)	53.2 (5.6)	36.7 (3.9)	46.0 (9.5)
Fat mass (kg)	17.0 (5.5)	16.7 (5.0)	16.9 (5.3)

The data are presented as means (standard deviation).

^a^Skeletal muscle index (kg/m^2^) = skeletal muscle mass/height^2^.

### Single-variant association analysis and functional annotation of associated variants for SMI

After the imputation of the genotypes, the number of single nucleotide polymorphisms (SNPs) included in the GWAS was 6,391,983 in 2046 individuals. The genomic inflation factor (λ) was 1.009, and no population stratification was observed in our dataset using principal component analysis (PCA) and the QQ plot (Supplementary Figs. S1 and S2).

**Figure 1:**
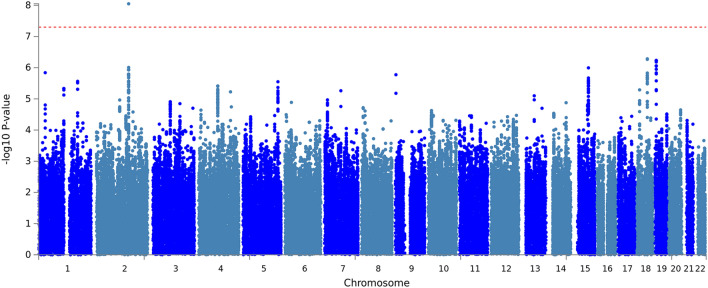
Manhattan plot of SNP-based GWAS for skeletal muscle index (SMI). The y-axis shows the -log_10_ p-values for SNPs in the GWAS. The horizontal red dotted line represents the threshold of genome-wide significance (*p* = 5 × 10^–8^). The manhattan plot was generated using Functional Mapping and Annotation of Genome-Wide Association Studies (FUMA) v1.3.6 (https://fuma.ctglab.nl).

The results of single-variant association analysis for the SMI showed a genome-wide significance (*p* < 5 × 10^–8^) on chr2q23 (Fig. 1). The strongest associated SNP, rs138684936 (β = 0.217, minor allele frequency = 0.212, *p* = 6.83 × 10^–9^), was located on the intron of the *NEB* gene, which encodes nebulin, a giant protein component of the cytoskeletal matrix that coexists with the thick and thin filaments within the sarcomeres of skeletal muscle. We also observed suggestive associations with rs2586725 (β = − 0.216, *p* = 5.20 × 10^–7^) near RP11-25O3.1 on chromosome 18 and rs8103412 (β = 0.137, *p* = 5.82 × 10^–7^) near *TNFSF9* on chromosome 19. However, most SNPs except the *FRK* gene identified in previous GWA studies^11,12,14,17,18^ for lean body mass were not significant in our sample (Supplementary Table S1). Variants in the *FTO* gene, rs17817964 and rs9936385, showed *p*-values of 0.066 and 0.065, respectively.

We used FUMA, a tool to functionally map and annotate GWAS results, and extracted significant independent SNPs and 87 candidate SNPs, which were in linkage disequilibrium (LD, r^2^ > 0.6) with the independent lead SNPs. Of all the candidate SNPs, 71 were in intronic regions, seven were in exonic regions, five were in UTR3, three were in intergenic, and one was in the ncRNA intronic region, and they mapped to nine genes (Supplementary Table S2). Most SNPs were also enriched for chromatin state 4, implying strong transcription. In the exonic regions, six SNPs were non-synonymous variants on *NEB* or *RIF1* genes (Table 2). Among them, the SNPs with high combined annotation dependent depletion (CADD) scores were rs2288210 (CADD = 20) on exon 114 of *NEB*, rs7575451 (CADD = 16.93) on exon 171 of *NEB*, and rs2444263 (CADD = 12.39) on exon 22 of *RIF1*, with GWAS *p*-values of 1.21 × 10^–4^, 5.50 × 10^–6^, and 6.11 × 10^–6^, respectively, in high LD (r^2^ > 0.7) with the lead SNP (rs138684936).

**Table 2:** Exonic variants in the genomic loci associated with the SMI and in LD (r^2^ > 0.6) with the independent genome-wide significant SNPs.

rsID	CHR	BP	Major/minor allele	MAF	gwasP	beta	r^2^	IndSigSNP	Gene	Exon	Exonic function	CADD	RDB	minChrState
rs2444263	2	152311570	A/G	0.244	6.11E−06	0.150	0.743	rs138684936	*RIF1*	22	Nonsynonymous	12.39	NA	4
rs2123465	2	152320118	A/G	0.242	6.11E−06	0.150	0.731	rs138684936	*RIF1*	30	Nonsynonymous	0.52	NA	3
rs2444257	2	152322095	T/A	0.238	6.13E−06	0.150	0.747	rs138684936	*RIF1*	30	Nonsynonymous	0.03	5	4
rs1065177	2	152331418	G/C	0.240	6.13E−06	0.150	0.759	rs138684936	*RIF1*	35	Nonsynonymous	3.52	5	2
rs7575451	2	152352843	G/C	0.249	5.50E−06	0.151	0.775	rs138684936	*NEB*	173	Nonsynonymous	16.93	6	4
rs2288210	2	152422076	G/C	0.224	1.21E−04	0.130	0.730	rs138684936	*NEB*	116	Nonsynonymous	20.00	7	4
rs6709886	2	152490219	G/A	0.227	6.59E−05	0.135	0.729	rs138684936	*NEB*	65	Synonymous	0.51	4	4

SNP p-values were computed using the linear regression model of additive allelic effects in PLINK (N = 2046 individuals). See “Methods” for the definition of independent significant SNPs (IndSigSNP). *rsID* rs number of the SNP, *CHR* chromosome, *BP* base-pair position on hg19, *MAF* minor allele frequency, *gwasP* SNP p-value for the SMI GWAS, *CADD* combined annotation dependent depletion (CADD) computed based on 63 annotations. The higher the score, the more deleterious the SNP, *RDB* RegulomeDB score, which is a categorical score (from 1a to 7). 1a is the highest score for SNPs with the most biological evidence to be a regulatory element, *minChrState* the minimum 15-core chromatin state across 127 tissue/cell types.

**Figure 2:**
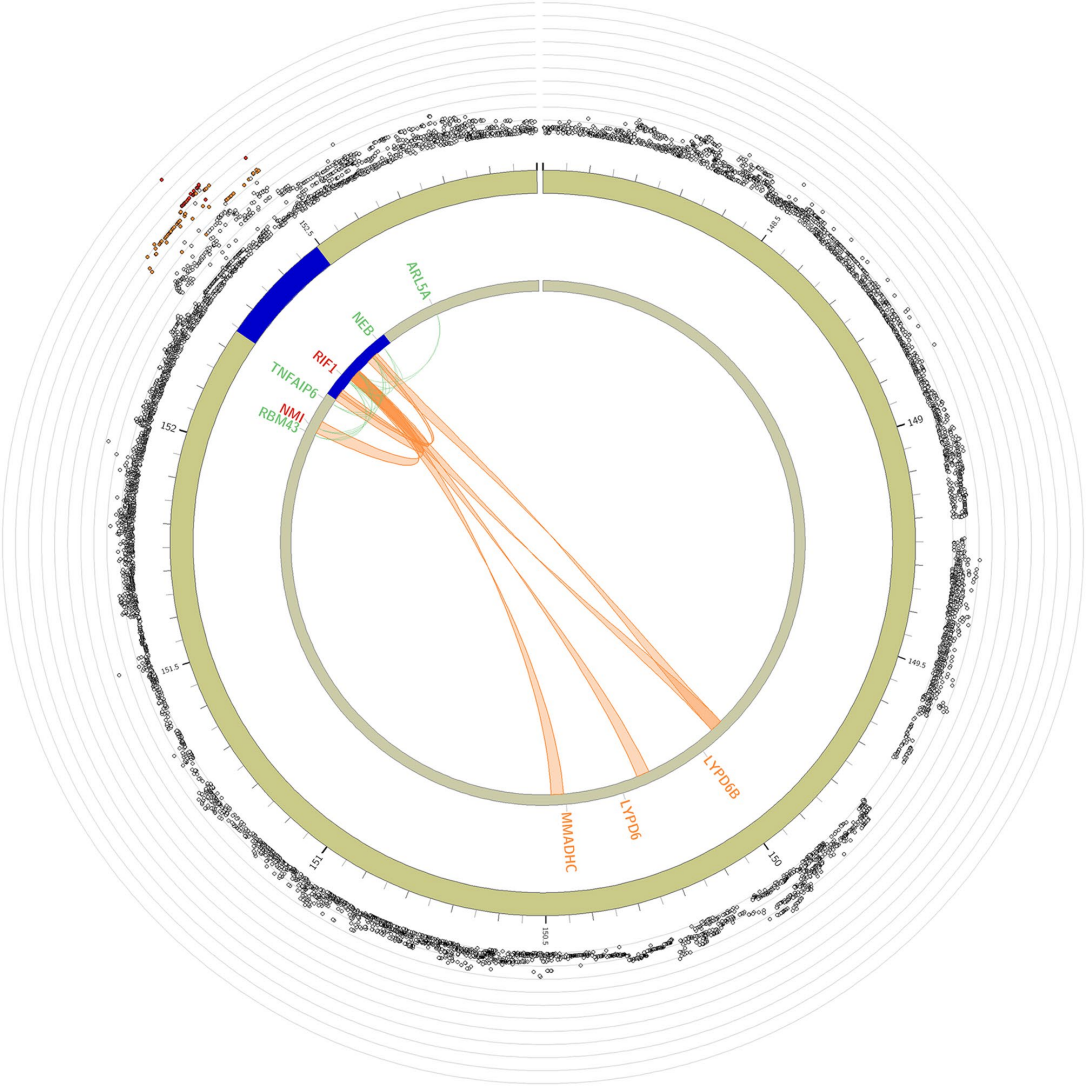
Cross-locus interactions for genomic regions associated with SMI. Circos plot showing genomic risk loci on genes on chromosome 2 implicated by eQTL (green), chromatin interaction (CI; orange), or implicated by both eQTL and CI mapping (red). The outer layer shows a Manhattan plot containing the -log10-transformed p-value of each SNP in the GWAS. The circos plot was generated using FUMA v1.3.6 (https://fuma.ctglab.nl).

**Figure 3:**
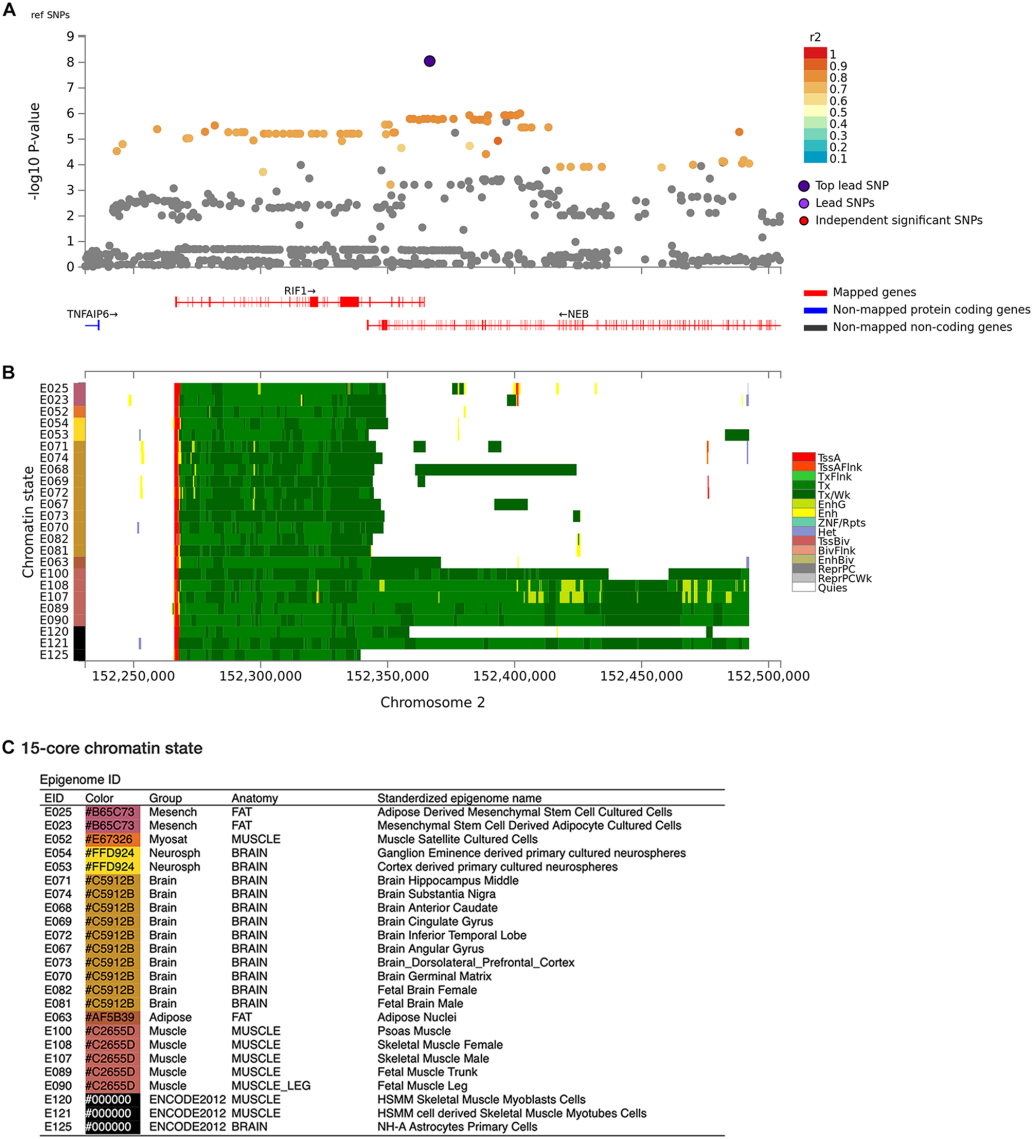
Regional plot and chromatin state on chromosome 2. (**A**) Regional plot of rs138684936 and SNPs in high LD (r^2^ > 0.6) with the lead SNP. (**B**) Chromatin 15 state in fat, muscle, and brain tissues. (**C**) Legend for the 15-core chromatin state. The regional plot was generated using FUMA v1.3.6 (https://fuma.ctglab.nl).

To link the candidate SNPs to genes, we used three gene-mapping strategies, positional, expression qualitative trait loci (eQTL), and chromatin interaction mapping. Based on our GWAS results, positional gene mapping annotated SNPs to two genes by genomic location and functional annotation, eQTL mapping matched *cis*-eQTL SNPs to six genes whose expression levels they influence in one or more tissues, and 3D chromatin interaction mapping mapped SNPs to five genes based on chromatin interaction such as HiC (Fig. 2, Supplementary Tables S3, S4, and S5). The *RIF1* gene was implicated by all three mapping strategies, and the *NEB* gene was prioritized by both positional and eQTL mapping. *RIF1* was mapped by eQTLs in several tissue types such as adipose subcutaneous, brain cortex, and esophagus muscularis. We found that our associated SNPs in *NEB* were not significant eQTLs in skeletal muscle even though *NEB* is predominantly expressed in skeletal muscle with the highest median transcripts per million (TPM = 846.4) from the GTEx v8 database (Supplementary Fig. S3). However, we found significant enrichment of alternative splicing QTL (sQTL) for *NEB,* and the 74 SNPs in or near *NEB* identified by the current GWAS were sQTLs in skeletal muscle and the atrial appendage from the GTEx (Supplementary Table S6). The lead SNP rs138684936 and variants in LD (r^2^ > 0.6) contained the active transcription start site (TSS) of *RIF1* and most variants overlapped with transcription and enhancer marks located in the regulatory regions for fat, muscle, and brain tissues (Fig. 3).

### Phenome-wide association study

The lead SNP associated with SMI and exonic SNPs in the Table2 were further investigated using a PheWAS (phenome-wide association study) at the GWAS ATLAS resource. These SNPs were associated with multiple traits belong to the metabolic and immunological domains (Supplementary Table S7). Generally, the pleiotropic effects were caused by one SNP associated with multiple correlated phenotypes. For example, the rs2444263 was significantly associated with estimated glomerular filtration rate (eGFR) and impedance of arm, age at menopause, and impedance of whole body, and trunk fat percentage, etc. (Bonferroni corrected p < 0.05, Supplementary Table S7). Genetic correlations were also found between the multiple traits associated with our top SNPs (Supplementary Fig. S4).

### Gene, and gene set, and tissue-expression analysis for SMI using MAGMA

We performed a gene-based association analysis using all SNPs in the GWAS. Table 3 and Supplementary Fig. S5 show the ten top-ranked genes associated with the SMI (nominal *p* < 1 × 10^–6^). Of the total 18,870 genes, only *TNFSF9* was significantly associated with the SMI (Bonferroni *p* < 0.05), but we also observed suggestive associations with *NEB* (nominal *p* = 6.53 × 10^–5^) and *RIF1* (nominal *p* = 5.47 × 10^–5^).

**Table 3:** Genes associated with the SMI by gene-based association analysis using MAGMA.

Gene	CHR	START	STOP	NSNPS	ZSTAT	*P*	Bonferroni *Q*
*TNFSF9*	19	6511010	6555931	27	5.058	2.12E−07	4.00E-03
*CT62*	15	71382583	71427833	35	4.3462	6.93E−06	1.31E-01
*RIC8B*	12	107148373	107303090	139	4.0769	2.28E−05	4.31E-01
*RFX4*	12	106956685	107176581	275	4.0594	2.46E−05	4.64E-01
*RAB7A*	3	128424965	128553639	232	4.0518	2.54E−05	4.80E-01
*DRD4*	11	617293	660706	126	4.0239	2.86E−05	5.40E-01
*RP11-144F15.1*	12	106869736	107188696	459	3.8714	5.41E−05	1
*NEB*	2	152321850	152611001	704	3.8254	6.53E−05	1
*EDEM3*	1	184639365	184744047	419	3.7221	9.88E−05	1
*OR9G4*	11	56490303	56531287	162	3.712	1.03E−04	1

Input SNPs were mapped to 18,870 protein-coding genes. CHR, chromosome; START/STOP, the annotation boundaries of the gene on that chromosome; NSNPS, the number of SNPs annotated to that gene that was found in the data; ZSTAT, the Z-value for the gene based on its (permutation) p-value; *P*, the gene *p*-value, using asymptotic sampling distribution; Bonferroni P, Bonferroni adjusted *q*-value (significant threshold = 0.05/18,870 = 2.65E−6).

The Multi-marker Analysis of GenoMic Annotation (MAGMA) gene-set analysis integrated within FUMA was performed for curated gene sets and gene ontology (GO) terms obtained from MsigDB. Using the gene-based *p*-values, we next performed gene-set analysis using a total of 15,480 gene sets. The top-ranked biological processes were *regulation of multicellular organism growth* (from GO), *presynaptic modulation of chemical synaptic transmission* (GO), and *cranial skeletal system development* (GO), of which only the gene set for the *regulation of multicellular organism growth* was statistically significant after correcting for multiple comparisons (Bonferroni *p* = 0.036, Table 4). Supplementary Table S8 shows a detailed association of the genes and the number of SNPs mapped to the gene in the gene set for the *regulation of multicellular organism growth.*

**Table 4:** Top ten ranked pathways associated with skeletal muscle mass.

Gene sets	NGENES	BETA	BETA STD	SE	*P*	Bonferroni *Q*
GO_bp: regulation of multicellular organism growth	68	0.50	0.03	0.11	2.34.E−06	3.62E−02
GO_bp: presynaptic modulation of chemical synaptic transmission	13	1.15	0.03	0.28	2.62.E−05	4.06E−01
GO_bp: cranial skeletal system development	66	0.42	0.03	0.11	4.69.E−05	7.26E−01
GO_bp: regulation of organelle organization	1175	0.10	0.02	0.03	6.70.E−05	1.00
GO_bp: embryonic cranial skeleton morphogenesis	45	0.45	0.02	0.12	1.21.E−04	1.00
GO_bp: proline transport	9	1.06	0.02	0.29	1.24.E−04	1.00
GO_bp: epithelial structure maintenance	28	0.61	0.02	0.17	1.48.E−04	1.00
GO_bp: positive regulation of synaptic vesicle transport	11	0.98	0.02	0.28	2.38.E−04	1.00
GO_bp: apoptotic mitochondrial changes	112	0.28	0.02	0.08	2.54.E−04	1.00
GO_mf: norepinephrine binding	5	1.25	0.02	0.37	3.06.E−04	1.00

For MAGMA analysis, 15,480 gene sets (curated gene sets, 5497; gene ontology terms, 9983) from MsigDB v7.0 were used.

*GO* gene ontology, *GO_bp* GO biological process, *GO_mf* GO molecular function, *NGENES* the number of genes in the data that was in the set, *BETA* the regression coefficient of the variable, *BETA STD* the semi-standardized regression coefficient, corresponding to the predicted change in Z-value given a change of one standard deviation in the predictor gene set/gene covariate (i.e., BETA divided by the variable’s standard deviation), *SE* the standard error of the regression coefficient, *P* *p*-value, *Bonferroni* *Q* Bonferroni adjusted *q*-value.

**Figure 4:**
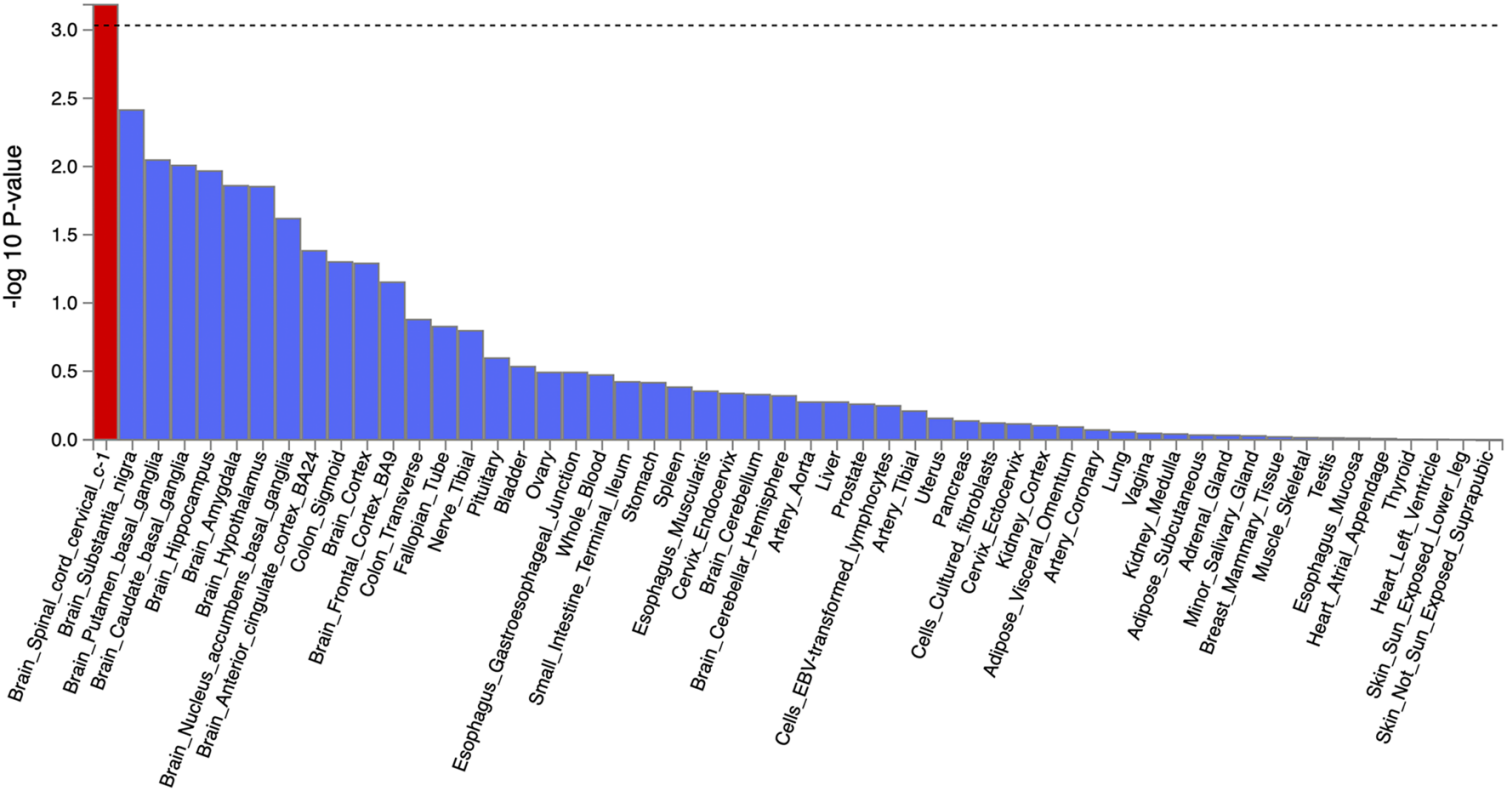
MAGMA tissue expression analysis of 54 tissue types from the genotype-tissue expression (GTEx) database. The bar plot was generated using FUMA v1.3.6 (https://fuma.ctglab.nl).

To identify tissue specificity of the SMI, we performed tissue expression analysis by MAGMA integrated within FUMA to test the relationships between tissue-specific gene expression profiles and genes associated with SMI in 54 tissue types obtained from the Genotype-Tissue Expression (GTEx) Project. The SMI was significantly associated with genes expressed in the brain spinal cord cervical c-1 region (Bonferroni *q* = 0.039) (Fig. 4.).

## Discussion

Here, we report the novel associations of skeletal muscle index (SMI) with loci in *NEB* and *RIF1* on chr2q23 in Koreans. The strongest association among the significant SNPs was located in the intron of the *NEB* and *RIF1* genes with the lowest *p*-value of 6.83 × 10^–9^. The *NEB* gene encodes for nebulin, a giant protein component of the cytoskeletal matrix that coexists with the thick and thin filaments within the sarcomeres of skeletal muscle^22^. Its critical role in muscle function became apparent when mutations in *NEB* were associated with autosomal recessive nemaline myopathy, a disease characterized by generalized skeletal muscle weakness and the presence of electron-dense protein accumulations (nemaline rods) seen on patient muscle biopsy examination^23,24^. Although the important role of *NEB* for skeletal muscle is well known, to our knowledge, variants in *NEB* have not been reported in prior GWA studies of muscle-related phenotypes (whole-body or appendicular lean body mass) through a review of the GWAS catalog (https://www.ebi.ac.uk/gwas/genes/NEB). The protein isoform sizes vary from 600 to 800 kD due to alternative splicing that is tissue-, species-, and developmental stage-specific. Of the 183 exons in the *NEB* gene, exons 63–66, 82–105, 143–144, and 166–177 are key regions where alternative splicing occurs^25^. The alternatively spliced exons 166–177 express at least 20 different transcripts in the adult human tibialis anterior muscle alone. We found an association with the SMI in exons 65, 116, and 173 of *NEB*. Alternative splicing is a common mechanism used to create muscle proteins specific for different muscle types and muscles of different developmental stages^26,27^. Alternatively, spliced exons in the 3′ end of the gene, as well as in the central region, account for the broad isoform diversity of nebulin^28,29^. Extensive alternative splicing of NEB may explain the pathogenesis of muscle-related diseases.

Other associated variants were in the *RIF1* gene, which is located next to the *NEB* and the genes partially overlap each other. The replication timing regulatory factor 1 (*RIF1*) gene encodes a protein that shares homology with the yeast telomere-binding protein, repressor/activator protein 1 (RAP1) interacting factor 1. RIF1 is a highly conserved protein whose functions have diverged during the course of evolution from its primary role in telomere length maintenance to a broader role in DNA replication, DNA repair, and the maintenance of genomic integrity^30^. A number of studies have been conducted to evaluate telomere stabilization in skeletal muscle tissue, generally associated with aging and physical activity^31^.

Both *NEB* and *RIF1* genes are known to produce multiple transcript variants by alternate splicing. Alternative splicing of precursor mRNA is an essential mechanism to increase the complexity of gene expression and plays an important role in cellular differentiation and organism development^32^. Singh et al. reported that alternative splicing substantially contributed to muscle homeostasis in adults^33^. We found that the associated SNPs for the SMI were identified as sQTLs and the six nonsynonymous SNPs with high CADD scores were highly conserved, suggesting that they might be essential for the development and maintenance of skeletal muscle mass. We also identified pathways related to skeletal system development or skeleton morphogenesis associated with the SMI. Such a role was also supported by our extensive functional annotation, showing that rs138684936 and SNPs in high LD overlapped with potential regulatory regions for muscle, fat, and brain tissues.

It is also interesting to note that the *TNFSF9* gene was the most significant in the gene analysis using MAGMA, and not the *NE*B or *RIF1* genes where the top SNP resided, although *NEB* showed a suggestive significant *p*-value in MAGMA. The results in the mapped genes from FUMA and the gene-based test using MAGMA may be different because the FUMA uses only significant SNPs and SNPs in LD with the significant SNPs, but the MAGMA uses all SNPs for the gene-based test. TNF receptor superfamily member 9 (*TNFRSF9*), also known as *CD137,* is implicated in inflammatory diseases such as atherosclerosis and Crohn’s disease.

Tissue expression analysis of 54 tissue types showed significant associations between brain spinal cord cervical c-1 and the SMI, but not skeletal muscle. The spinal cord has been suggested to be associated with aging^34^. Although many other measures of corticospinal communication appear unaffected by aging, the excitatory postsynaptic potential (EPSP) in spinal motoneurons, which is induced by fast-conducting descending volleys, show a linear decline with age^35,36^. The number of spinal motor neurons declines with age, which is associated with an increase in the number of astrocytes and apparent alterations in the neuronal dendritic networks^37^. These changes may cause reductions in muscle mass, strength, and performance with aging^34^. In the current study, our subjects are relatively young (mean/SD, 39.3/8.9 years) because this cohort comprised middle-aged office workers and their spouses^38^, and only 11 individuals were elderly (> 65 years). Janssen et al*.* have reported that men had significantly greater skeletal muscle mass than women with greater losses of skeletal muscle mass with aging^20^. Age-associated loss of muscle mass appears inevitable and is likely the most significant contributing factor to the decline in muscle strength^39^. Although we could not perform age-stratified analyses due to small sample size, the association of the *RIF* gene in the current study might support a link between skeletal muscle mass and aging.

It is important to note that the candidate loci were not consistent with previously reported loci for lean body mass. The SNPs reported in previous GWA studies^11,12,17,18^, including the study by Zillikens et al*.*^14^ for lean body mass, were not significant in our sample. Although their study was the largest GWAS on lean body mass, most of the participants were Europeans and the main results showed that both the discovery and replication sets were the results of Europeans, although an Asian data set was included^14^. They performed a GWAS using a European population whose body composition is known to be different from that of Asians. Usually, Asian people have less skeletal muscle than Europeans^20,21^. Additionally, lean body mass, mainly skeletal muscle mass, was adjusted with height in Zillikens’ study, not with height squared. As skeletal muscle mass is largely influenced by body mass, the European and Asian working group for sarcopenia corrected the skeletal muscle mass using height squared^7,8^. The different adjustments may contribute to the different results between our study and the previous trial. In the GWAS catalog of lean body mass (https://www.ebi.ac.uk/gwas/efotraits/EFO0004995), there was no GWA study where both the discovery samples and the replication samples were East Asian. Furthermore, the rs138684936 that showed the strongest signal in this study has > 20% minor allele frequency in East Asian including our results, while the frequency of the allele is immensely rare in most European population (0.1–0.8%) or low in African (3%) based on the Genome Aggregation Database (gnomAD; https://gnomad.broadinstitute.org). Therefore, further studies using East Asian samples are needed to confirm the observed associations for the *NEB* and *RIF1* genes.

Interestingly, we observed the pleiotropic effect of the top SNPs using PheWAS. The SNPs significantly associated with metabolic phenotypes (eGFR, impedance measures of body composition, body fat ratio, etc.), immunological phenotypes (mean corpuscular hemoglobin concentration, etc.), and psychiatric phenotypes (frequency of tiredness, smoking, alcohol, etc.), indicating the multiple phenotypes may be genetically correlated with SMI.

Several limitations of the current study should be discussed. First of all, we did not confirm the associations in independent cohorts. Since the current study evaluated skeletal muscle mass by BIA, replication data should have BIA data but there are scanty GWAS data samples with BIA data. Without replication, the limited number of subjects available for analysis limited the value of the results. Our results did not support the associations reported in previous GWA studies. More studies using independent cohorts in East Asian populations are needed to confirm our results because there are few GWA studies in East Asians. However, the population in this study was Korean, so the generalization of our findings to other ethnicities, even for East Asians, is limited. Second, the definition of skeletal muscle mass, the largest component of lean body mass, was not identical across all previous studies, which may introduce inconsistencies into the results. Previous GWA studies were performed with lean body mass^11–14^, which consisted of skeletal muscle mass, bone, skin, and connective tissue^40^. Actually, skeletal muscle mass cannot be measured exactly, which was estimated based on lean body mass measured with DEXA or BIA in the clinical situation. As the European and Asian working group published the definition of sarcopenia in which skeletal muscle mass was divided by height squared^7,8^, we used their methods in this study. Finally, the functional annotation should be underlined as only predictive, and the exact effect of the specific mutation should be verified in functional studies. Nevertheless, the combined strategies of functional annotation and gene-mapping provide extensive information on the likely consequences of relevant genetic variants and suggest a rich set of plausible gene targets and biological mechanisms for functional follow-up^41^.

In conclusion, we identified a new genetic locus on chromosome 2 for skeletal muscle mass with genome-wide significance, at least in Koreans. The current results shed light on the mechanism of skeletal muscle mass and urge further studies in East Asians to elucidate the pathophysiology of low skeletal muscle mass.

## Methods

### Subjects

The study population was comprised of a subset of Kangbuk Samsung Cohort Study (KSCS) participants and consisted of men and women aged 18 years or older who underwent annual or biennial health examinations^38^. After sample quality control for GWAS analysis, the remaining 2046 subjects consisted of 1150 men and 896 women aged 20–69 years.

### Anthropometric measurements

Data on demographic characteristics, smoking status, alcohol history, degree of physical activity, and history of hypertension, hyperlipidemia, and diabetes mellitus were collected by the examining physicians using standardized self-administered questionnaires. The individuals with smoking history were categorized into never, former, or current smokers. The individuals with alcohol consumption over 20 g/day were defined as heavy drinkers. The degree of physical activity was evaluated using the International Physical Activity Questionnaire Short Form. Regular physical activity was defined as vigorous exercise more than three times/week for > 20 min per session or moderate exercise as more than five times/week for > 30 min per session.

The patient demographic data, specifically age, height and weight, and anthropometric data, including SMI, skeletal muscle mass, total muscle mass, fat mass, BMI, and waist circumference, were reviewed. Height, weight, and body composition were measured with a multi-frequency BIA by trained nurses while the subjects wore lightweight hospital gowns and no shoes. The BIA had 8-point tactile electrodes (InBody 720, Biospace Co., Seoul, Korea) and was previously validated for reproducibility and accuracy for body composition^9^. Appendicular skeletal muscle mass was estimated based on appendicular lean body mass measured with the BIA and divided by height squared to derive the skeletal muscle index (SMI, kg/m^2^), based on the recommendation of the Asian Working Group for Sarcopenia^7^ to use height-squared adjusted skeletal muscle mass, and a recent report demonstrating that the height-squared adjusted skeletal muscle mass was better correlated with muscular function than body weight-adjusted skeletal muscle mass^42^. BMI was calculated as BMI (kg/m^2^) = weight (kg)/height^2^ (m^2^).

### Genome-wide association analysis

Genotyping was performed with the Illumina Infinium HumanCore BeadChips 12v1 kit (Illumina Inc., San Diego, CA, USA). In pre-imputation quality control (QC), SNP quality control procedures were conducted to eliminate ineligible SNPs (SNPs from mitochondria or X or Y chromosome, genotyping rate < 0.95, Hardy–Weinberg Equilibrium (HWE) *p-*value < 10^–6^, and minor allele frequency (MAF) < 0.01), following which, 226,706 autosomal SNPs remained. Sample quality control for GWAS analysis was performed on the raw samples, in which 62 ineligible subjects were eliminated (missing rate > 0.04, mean heterozygosity >  ± 3 SD, individuals from the same family, and unmatched sex). Imputation was conducted using reference panels from 1000 Genomes Phase 3 (v5) in the Michigan Imputation Server using Minimac4 (https://imputationserver.sph.umich.edu/index.html). Post-imputation cutoffs were applied, which included MAF > 0.01, imputation quality (R^2^) > 0.6, HWE *p*-value > 10^–6^, and SNP call rate > 0.98. The associations between GWAS SNPs and the SMI were analyzed with PLINK 1.90 beta software (https://www.cog-genomics.org/plink/1.9/). Linear regression analysis for the SMI was performed with PLINK statistical software after adjusting for the effects of age, sex, and principal component (PC)1, PC2, and PC3.

### Functional annotation

Functional annotation was conducted with SNP2GENE implemented in FUMA (v1.3.6)^43^. The FUMA platform was designed for prioritization, annotation, and the interpretation of GWAS results. As the first step, significant, independent SNPs in the GWAS summary statistics were identified based on their *p*-values (*p* < 5 × 10^–8^) and independence from each other (r^2^ < 0.6 in the 1000G phase 3 EAS reference) within a 250 kb window. After that, the lead SNPs were identified in the significant, independent SNPs, which were independent of each other (r^2^ < 0.1). SNPs that were in LD with the identified independent SNPs (r^2^ ≥ 0.6) within a 250 kb window were selected as candidate SNPs and taken forward for further annotation.

FUMA annotates candidate SNPs in genomic risk loci based on functional consequences on genes (ANNOVAR)^44^, CADD score^45^, potential regulatory functions (RegulomeDB scores, RDB)^46^, the effect on gene expression using eQTL of different tissue types (GTEx v8)^47^, and 3D chromatin interactions from Hi-C experiments of 21 tissues/cell types, also embedded in the FUMA platform. The CADD score is the score of the deleteriousness of the SNPs. A score of 12.37 is the suggested deleterious threshold and higher scores are more deleterious. A CADD score of ≥ 10 indicates a variant predicted to be among the top 10% most deleterious substitutions involving the human genome, a score of ≥ 20 indicates a variant among the top 1% most deleterious, and so forth^45^. Genes were mapped using positional mapping based on ANNOVAR annotations and maximum distance between SNPs and genes (default 10 kb), eQTL mapping, and 3D chromatin interaction mapping. Only significant eQTLs were used by default (FDR < 0.05). Chromatin interaction mapping was performed with significant chromatin interactions (defined as FDR < 1 × 10^–6^). We also used GTEx Analysis Release V8 (dbGaP Accession phs000424.v8.p2) to investigate splicing QTL (sQTLs) for the SNPs in different tissue types on the GTEx Portal (https://gtexportal.org/).

### Phenome-wide association studies

We verified the association between the lead variant and exonic variants in high LD (r^2^ > 0.6) with the lead variant and a wide range of phenotypes. We used the database contains 4756 GWAS from 473 unique studies across 3302 unique traits and 28 domains at the GWAS ATLAS resource (https://atlas.ctglab.nl/PheWAS)^48^. The number of curated phenotypes and the significance threshold were 28 for rs138684936 (*p* < 1.79 × 10^–3^, 0.05/28), 277 for rs2444263 (*p* < 1.81 × 10^–4^, 0.05/277), 278 for rs2123465 (*p* < 1.80 × 10^–4^, 0.05/278), 168 for rs2444257 (*p* < 2.98 × 10^–4^, 0.05/168), 172 for rs1065177 (*p* < 2.91 × 10^–4^, 0.05/172), 167 for rs7575451 (*p* < 2.99 × 10^–4^, 0.05/167), 172 for rs2288210 (*p* < 2.91 × 10^–4^, 0.05/172), and 127 for rs6709886 (*p* < 3.94 × 10^–4^, 0.05/127), respectively. Genetic correlations were computed for pair-wise GWASs with criteria as suggested previously using LD Score regression (LDSC)^49^ at the GWAS ATLAS^48,50^.

### Gene-based and gene set enrichment analyses, and gene property analysis for tissue specificity

The gene-based analysis was conducted with MAGMA v1.07^51^ with default settings implemented in FUMA. For FUMA, 15,480 gene sets (curated gene sets, 5497; GO terms, 9983) from MsigDB v7.0 were used. In the MAGMA gene-based analysis, the SNPs are mapped to protein-coding genes if they are located in the gene, and the resulting SNP *p*-values are combined into a gene test-statistic using the SNP-wise mean model. Bonferroni’s correction was performed for all tested gene sets. To identify tissue specificity of the phenotype, FUMA performs MAGMA gene-property analyses to test the relationships between tissue-specific gene expression profiles and disease-gene associations.

### Statement of ethics

The Institutional Review Board of Kangbuk Samsung Hospital approved this study (IRB No. 2020-07-048). Written informed consent was obtained from all participants. The process of this research was conducted according to relevant guidelines and regulations.

## Supplementary Information

Supplementary Information 1.

Supplementary Information 2.
