# Supplementary material for: Variants in NEB and RIF1 genes on chr2q23 are associated with skeletal muscle index in Koreans: genome-wide association study
Source: Sci Rep. 2021 Mar 5;11:2333. doi: 10.1038/s41598-021-82003-y (PMC7935852; doi:10.1038/s41598-021-82003-y)
Supplement: Supplementary file 1 — Supplementary Information 1. [file 41598_2021_82003_MOESM1_ESM.docx]

**Supplemental Information**

**Variants in *NEB* and *RIF1* genes on chr2q23 are associated with skeletal muscle index in Koreans: Genome-wide association study**

Kyung Jae Yoon, Youbin Yi, Jong Geol Do, Hyung-Lae Kim, Yong-Taek Lee, Han-Na Kim


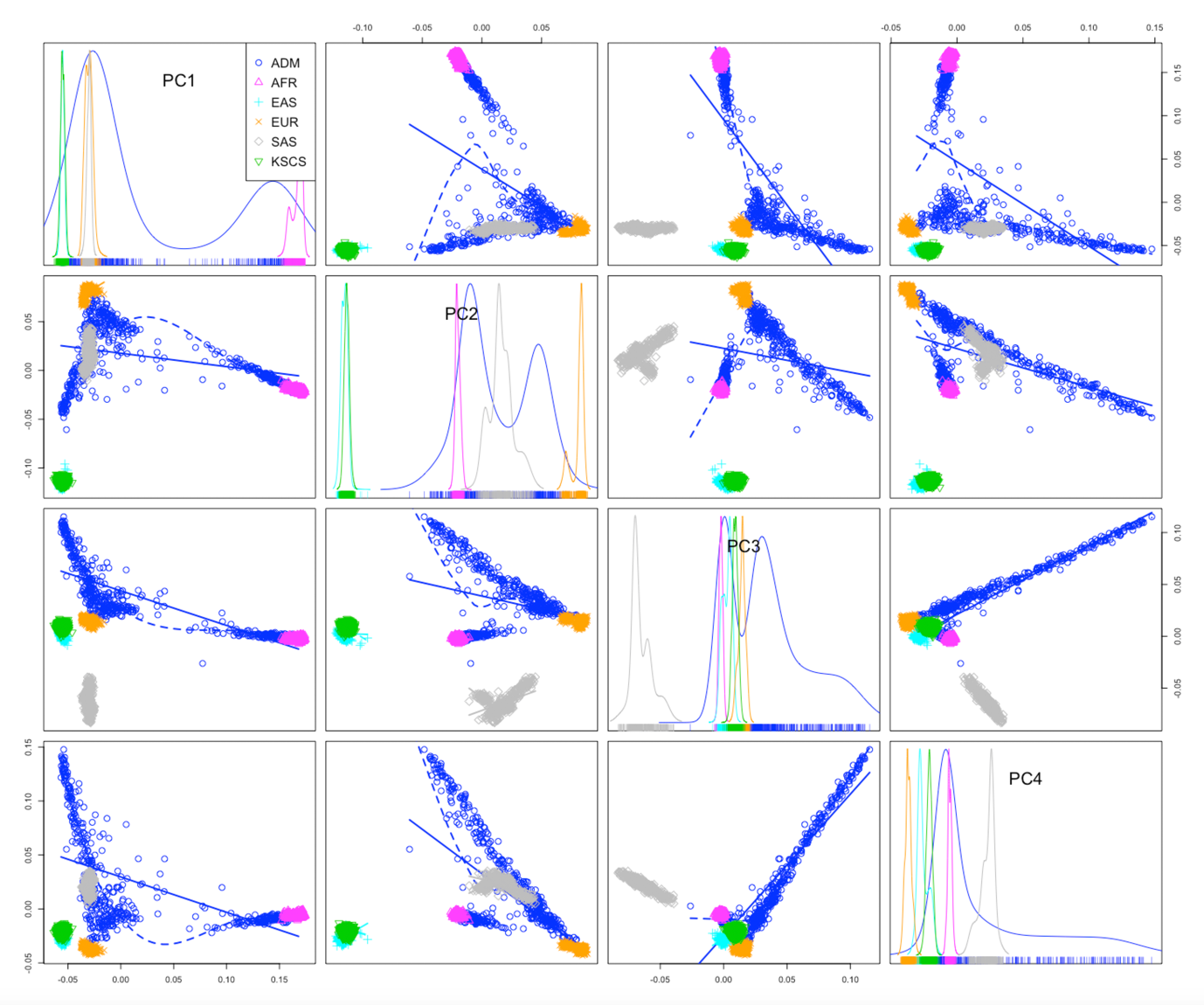


**Supplementary Figure S1. PCA plot.** ADM, admixed American; AFR. African; EAS, East Asian; EUR, European; SAS, South Asian; KSCS, Kangbuk Samsung Cohort Study (current study)


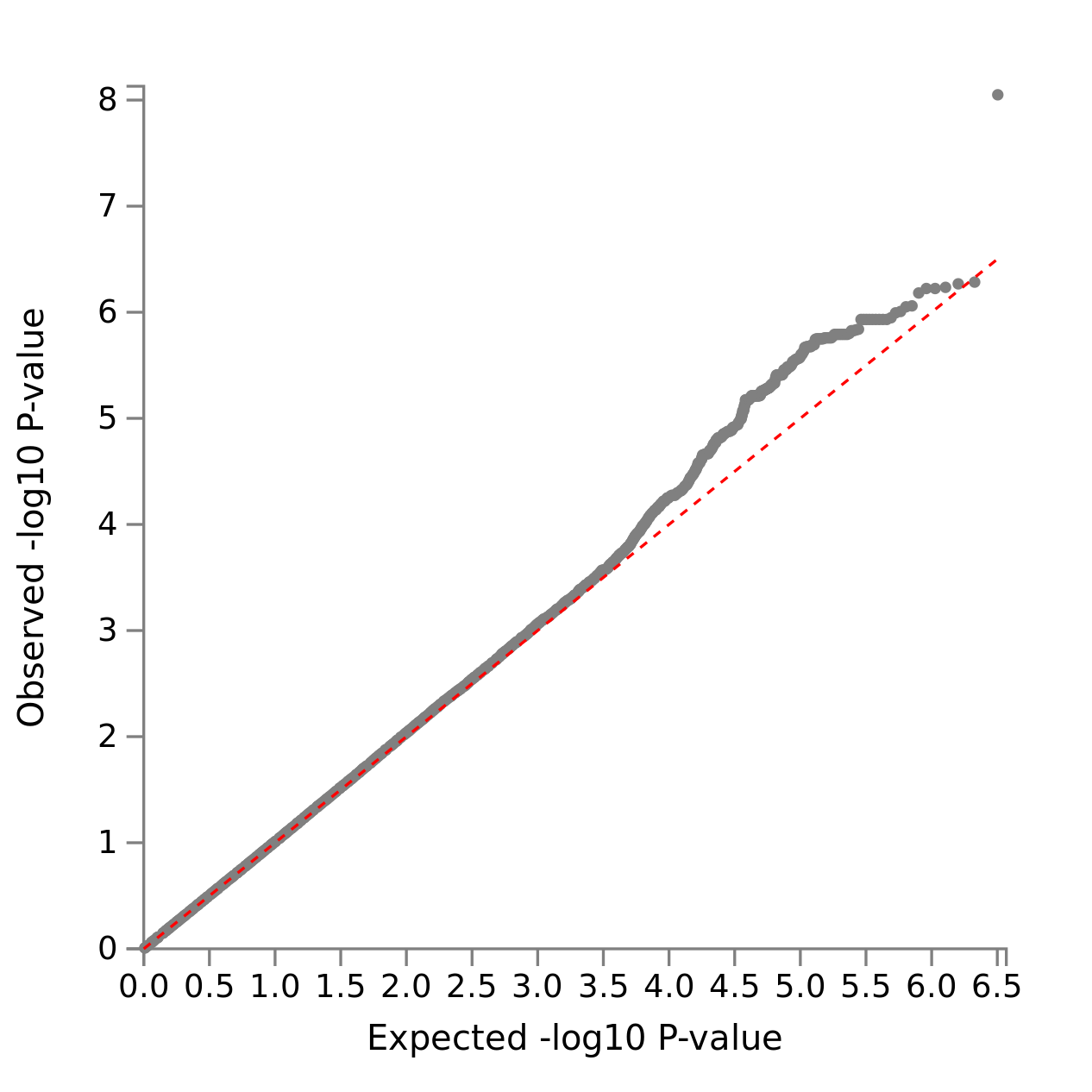


**Supplementary Figure S2. QQ plot**


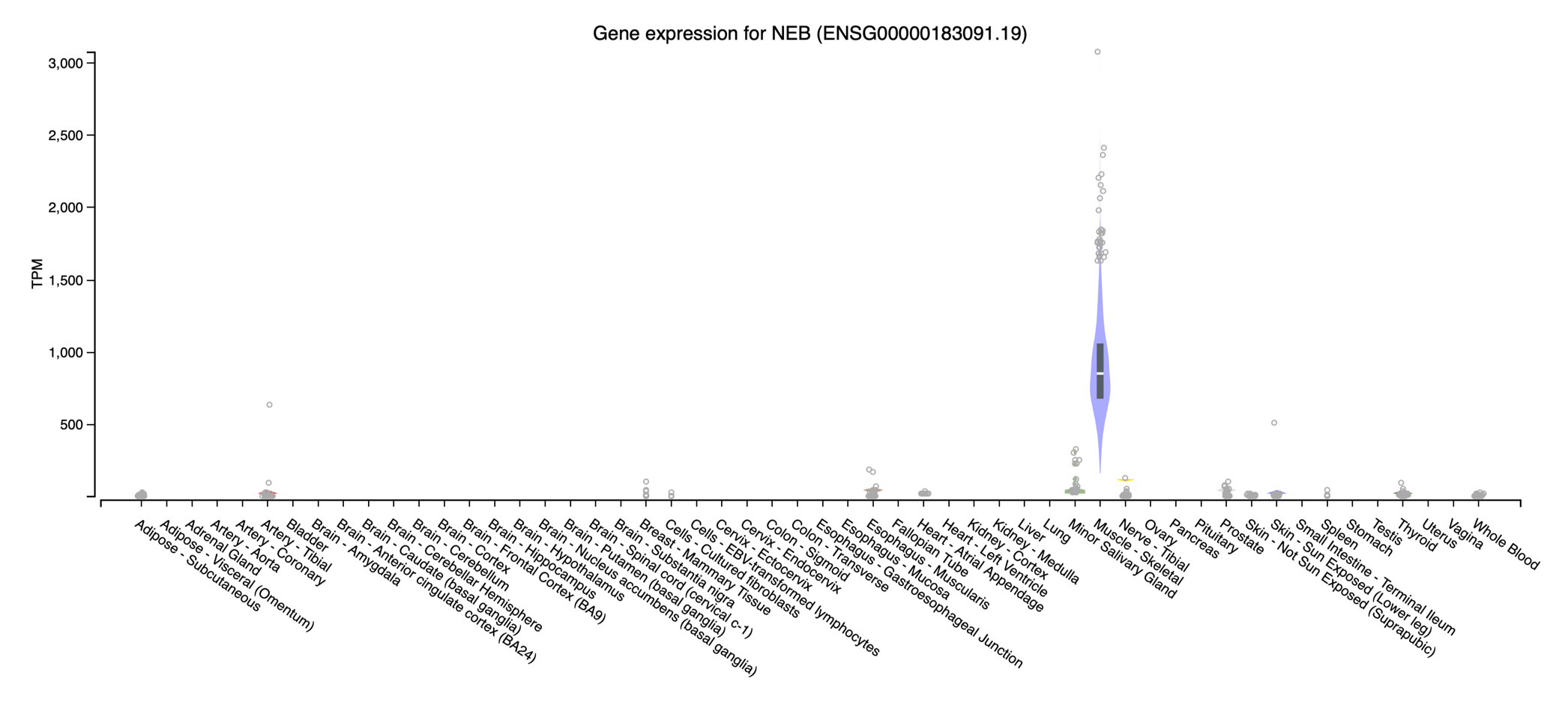


**Supplementary Figure S3. Gene expression for NEB in the multi-tissues from GTEx Portal**

**Supplementary Figure S4. Genetic correlation (***r_g_*) **within and between domains which showed significant associations in the PheWAS for the top SNPs of SMI GWAS.** Heatmap of proportion of trait pairs with significant *r_g_* (upper right triangle) and average |*r_g_*| for significant trait pairs (lower left triangle) between domains. Stars denote the pairs of domains in which the majority (>50%) of significant *r_g_* are positive or negative.
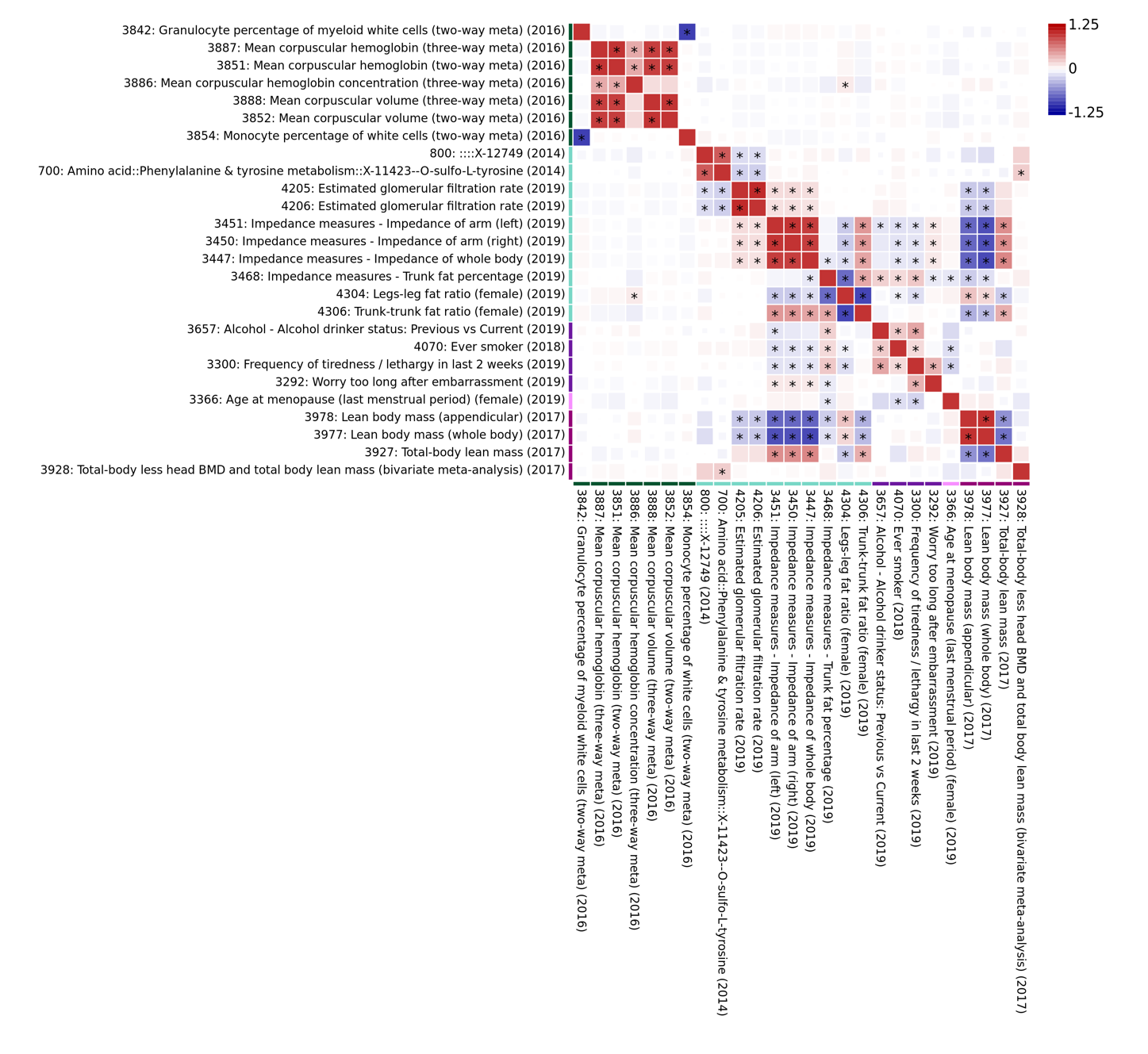
Plot was depicited at the GWAS ATLAS resource (https://atlas.ctglab.nl/PheWAS).


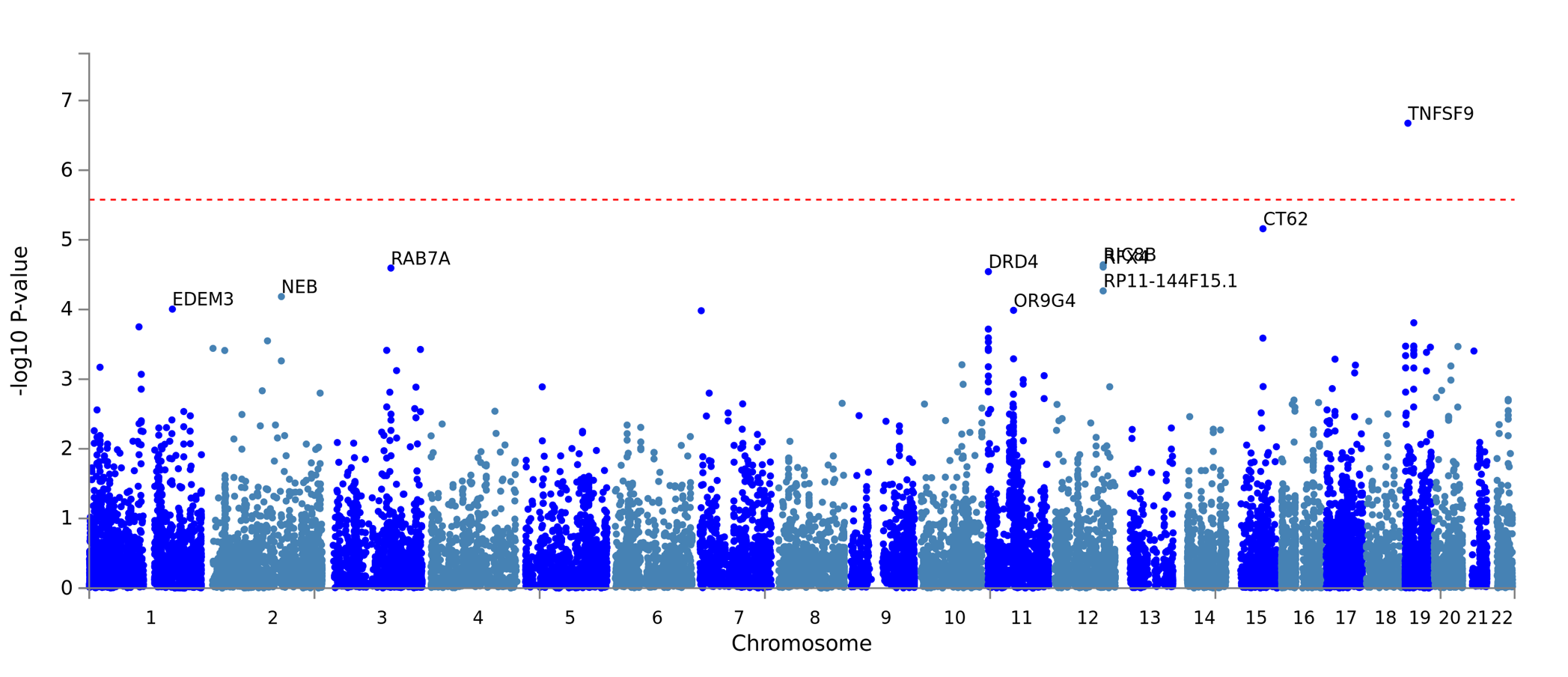


**Supplementary Figure S5. Manhattan plot of gene-based analysis.** This is a plot of the p-values of 18,870 genes computed by MAGMA based on GWAS summary statistics. The top 10 genes with the lowest p-values were annotated. The red dotted line indicates the Bonferroni threshold at α = 0.05.
